# Isotopic Radiolabeling of the Antiretroviral Drug [^18^F]Dolutegravir for Pharmacokinetic PET Imaging

**DOI:** 10.3390/ph15050587

**Published:** 2022-05-10

**Authors:** Marion Tisseraud, Sébastien Goutal, Thomas Bonasera, Maud Goislard, Delphine Desjardins, Roger Le Grand, Chris M. Parry, Nicolas Tournier, Bertrand Kuhnast, Fabien Caillé

**Affiliations:** 1 Université Paris-Saclay, Inserm, CNRS, CEA, Laboratoire d’Imagerie Biomédicale Multimodale Paris-Saclay (BioMaps), 91401 Orsay, France; marion.tisseraud@laposte.net (M.T.); sebastien.goutal@cea.fr (S.G.); maud.goislard@cea.fr (M.G.); nicolas.tournier@cea.fr (N.T.); bertrand.kuhnast@cea.fr (B.K.); 2 GSK Medicines Research Centre, Gunnels Wood Road, Stevenage SG1 2NY, UK; thomas.x.bonasera@gsk.com; 3 Université Paris-Saclay, Inserm, CEA, Center for Immunology of Viral, Auto-Immune, Hematological and Viral Diseases (IMVA-HB/IDMIT), Fontenay-aux-Roses, 92032 Paris, France; delphine.desjardins@cea.fr (D.D.); roger.legrand@cea.fr (R.L.G.); 4 ViiV Healthcare, 980 Great West Road, London TW8 9GS, UK; chris.2.parry@viivhealthcare.com

**Keywords:** fluorine-18, radiolabeling, dolutegravir, PET imaging

## Abstract

Deciphering the drug/virus/host interactions at infected cell reservoirs is a key leading to HIV-1 remission for which positron emission tomography (PET) imaging using radiolabeled antiretroviral (ARV) drugs is a powerful asset. Dolutegravir (DTG) is one of the preferred therapeutic options to treat HIV and can be isotopically labeled with fluorine-18. [^18^F]DTG was synthesized via a three-step approach of radiofluorination/nitrile reduction/peptide coupling with optimization for each step. Radiofluorination was performed on 2-fluoro-4-nitrobenzonitrile in 90% conversion followed by nitrile reduction using sodium borohydride and aqueous nickel(II) chloride with 72% conversion. Final peptide coupling reaction followed by HPLC purification and formulation afforded ready-to-inject [^18^F]DTG in 5.1 ± 0.8% (*n* = 10) decay-corrected radiochemical yield within 95 min. The whole process was automatized using a TRACERlab^®^ FX NPro module, and quality control performed by analytical HPLC showed that [^18^F]DTG was suitable for in vivo injection with >99% chemical and radiochemical purity and a molar activity of 83 ± 18 GBq/µmol (*n* = 10). Whole-body distribution of [^18^F]DTG was performed by PET imaging on a healthy macaque and highlighted the elimination routes of the tracer. This study demonstrated the feasibility of in vivo [^18^F]DTG PET imaging and paved the way to explore drug/virus/tissues interactions in animals and humans.

## 1. Introduction

Increasing evidence makes remission an achievable goal for new human immunodeficiency virus (HIV-1) therapies [1,2,3]. Combined antiretroviral therapies (cART) suppress viral replication and drastically reduce morbidity and mortality [4]. However, despite the existence of a few patients capable of controlling HIV replication to undetectable levels (i.e., HIV controllers, <1% of the HIV-infected population) [2], cART do not eradicate infected cells, and viral reservoirs still remain [5]. Early control of these reservoirs together with the limitation of pharmacological sanctuaries where the exposure to cART is suboptimal [6] are among the key scientific challenges to make remission a reality for a majority of patients.

The whole-body distribution of the virus is a key parameter to predict the virus/host/drugs interactions. Indeed, localization of infected cell reservoirs and their interactions with both drugs and the tissue micro-environment remain poorly understood [7]. In this context, in vivo tomographic imaging technologies may be of considerable help to tackle anti-HIV therapy challenges [8,9,10]. In particular, positron emission tomography (PET) imaging is a sensitive and quantitative technique for in vivo kinetic modeling of drug distribution in tissues and accurate detection of molecular targets. Kinetics of drug diffusion and body distribution can theoretically be addressed in relevant animal models of the disease, in healthy volunteers, and in patients. New approaches to directly visualize metabolic pathways or viral replication in the host are rapidly emerging and have a strong potential for being translated in the near future into human clinical studies. Fluorine-18-labeled fluoro-D-deoxyglucose ([^18^F]FDG) is by far the most common radiotracer for PET imaging and has been used to monitor HIV infection before and after cART in the context of HIV-related inflammation [11]. However, [^18^F]FDG reacts to immune activation and is not specific for HIV reservoirs. More recently, ^18^F-labeled nucleoside derivatives have been developed aiming at imaging T-cell trafficking [12,13], but this strategy, which has never been applied to HIV imaging, is only an indirect visualization of immune activation. ImmunoPET imaging using radiolabeled antibodies offers promising perspectives in the path towards higher specificity for infection biomarkers [14]. First-in-human studies using antibodies labeled with copper-64 [15] and zirconium-89 [16] were conducted with HIV patients on and off ART treatment and demonstrated the feasibility and the relevance of PET imaging for HIV patient follow-up. From a therapeutic perspective, it is important to decipher the biodistribution of cART at remaining sanctuary sites and potential HIV-1 reservoirs. This could be achieved with PET imaging using radiolabeled antiretroviral drugs. This approach is currently in an early phase 1 clinical trial (NCT03174977) using fluorine-18-labeled raltegravir (MK-0518), the first integrase strand transfer inhibitor (INSTI) approved by the FDA. Dolutegravir (DTG, Tivicay^®^, ViiV Healthcare Limited, Brentford, UK) is another INSTI used in combination with other antiretroviral drugs to inhibit the integration of HIV proviral DNA and, hence, the production of new viral particles [17,18]. DTG offers better virological control than raltegravir [19] and is one of the preferred therapeutic options to treat HIV as recommended by the World Health Organization [20]. With two fluorine atoms on its structure, DTG is an excellent candidate for isotopic labeling with fluorine-18, i.e., radiolabeling without modification of the chemical structure and, therefore, without modification of biological properties. To assess the delivery of DTG to various critical tissue compartments and to understand the persistence of HIV-1 reservoirs to prolonged treatments, preclinical and clinical positron emission tomography (PET) using fluorine-18-radiolabeled DTG are of interest.

Herein we describe the different approaches that we explored to radiolabel DTG with fluorine-18 to finally afford a robust and reproducible automatized process to synthesize ready-to-inject [^18^F]DTG. We also report the first whole-body PET images and biodistribution of [^18^F]DTG in a healthy non-human primate.

## 2. Results and Discussion

### 2.1. Late-Stage Radiolabeling Approach

**Scheme 1:**
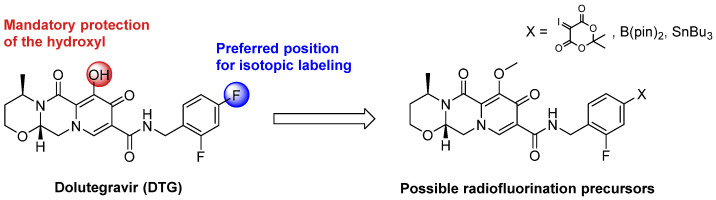
Possible precursors for late-stage isotopic radiofluorination of DTG with fluorine-18.

The most efficient strategy to radiolabel DTG with fluorine-18 would be a late-stage approach consisting of the incorporation of fluorine-18 at the final step of the synthesis on the aromatic core of DTG bearing two fluorine atoms (Scheme 1). However, this aromatic core is electron-rich, hampering radiofluorination by standard aromatic nucleophilic substitution (Sn_Ar_). Alternative approaches have been described using iodonium salts [21,22,23] or ylides [24,25,26] as precursors or by copper-catalyzed Cham-Lam-like fluorination using boronic acid [27,28], boronic ester [29,30,31,32], or trialkyltin precursors [33,34]. Late-stage isotopic radiolabeling of DTG was considered at the *para* position of the aromatic core (compared to the methyl amino group), as better radiochemical yields were depicted on this position compared to *ortho* [27,29,31,33]. Limitations of these fluorination methods were also described in the presence of unprotected alcohol or amine [29,31]. Therefore, the hydroxyl function of DTG was protected with a methyl moiety, the cleavage of such function being well described for DTG [35].

Labeling precursors for late-stage radiofluorination of DTG, i.e., iodonium ylide, boronic acid, and ester and tributyltin derivatives were synthesized in two steps (Scheme S1 in Supplementary Materials). Unfortunately, even if the boronic ester precursor was observed by liquid chromatography coupled with mass spectroscopy (LC-MS), it was too unstable to be isolated. The ylide precursor was synthesized but could not be isolated from its starting material and was unstable at 120 °C, whereas a minimal temperature of 150 °C is required to perform radiofluorination with this type of precursor [24,25]. Several attempts of copper-catalyzed radiofluorination using the tributyltin precursor were realized under the standard conditions described in the literature using the Cu(OTf)_4_Py_4_ complex [33], but incorporation of fluorine-18 was never observed (Scheme S2 in Supplementary Materials).

### 2.2. Three-Step Radiolabeling Approach

**Scheme 2:**
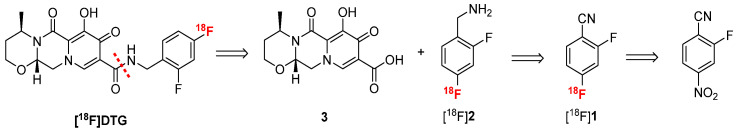
Retroradiosynthesis of [^18^F]DTG following the approach described for [^18^F]Raltegravir [36].

Considering the difficulties encountered for the late-stage labeling of DTG, the strategy described for raltegravir radiosynthesis was explored [36,37]. Mimicking the recent synthesis of dolutegravir described by Dietz et al. [38], [^18^F]DTG can be synthesized by peptide coupling between carboxylic acid **3** and radiolabeled difluorobenzylamine [^18^F]**2** (Scheme 2). Although the radiosynthesis of [^18^F]**2** has never been reported, *p*-[^18^F]fluorobenzylamine has been extensively described in the literature [39]. Following a similar approach, [^18^F]**2** could be synthesized by reduction of a nitrile moiety from [^18^F]**1** which could be obtained by radiofluorination of 2-fluoro-4-nitrobenzonitrile. This approach will benefit from the poor electron density of 2-fluoro-4-nitrobenzonitrile due to the nitrile moiety at the *para* position of the nitro group, which allows for the introduction of the fluorine-18 ion via a standard Sn_Ar_ reaction.

#### 2.2.1. Radiofluorination Optimization

**Scheme 3:**
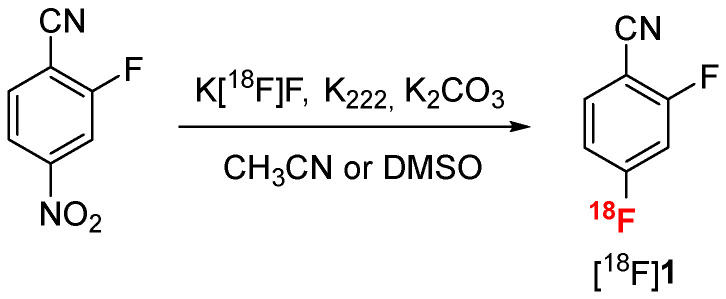
Radiofluorination of 2-fluoro-4-nitrobenzonitrile by Sn_Ar_.

Radiofluorination of 2-fluoro-4-nitrobenzonitrile was realized by Sn_Ar_ under standard conditions using the K [^18^F]F/K_222_ complex in acetonitrile (Scheme 3) [39]. A kinetic monitoring of the reaction using thin layer chromatography (TLC) analysis on silica gel (see Figure S12 in Supplementary Materials) revealed that performing the radiofluorination in acetonitrile at 95 °C for 5 min was the best compromise to reach a high conversion into [^18^F]**1** (96%) in a short time (Table 1, entry 1). However, acetonitrile, even at trace quantities, could be deleterious for the following step of nitrile reduction, and DMSO is usually preferred [40]. Radiofluorination in DMSO afforded [^18^F]**1** in 90% conversion (Table 1, entry 4), comparable with acetonitrile under the same conditions.

#### 2.2.2. Nitrile Reduction Optimization

Different approaches were explored for the nitrile reduction of compound [^18^F]**1** into [^18^F]**2** (Table 2). Lithium aluminium hydride (LAH) has been commonly used to reduce [^18^F]fluorobenzonitrile into [^18^F]fluorobenzylamine [39]. In our case, using LAH in THF afforded the desired [^18^F]**2** in 15 to 50% conversion depending on the reaction temperature (Table 2, entries 1 and 2) together with different side products. In particular, defluorination of [^18^F]**2** into *p*-[^18^F]fluorobenzylamine [^18^F]**2′** was observed. Indeed, hydrodefluorination of arenes in the *ortho* position of an electron-withdrawing group such as the nitrile moiety has been reported in the literature using metal hydrides [41,42]. Because of their structure similarity, [^18^F]**2** and [^18^F]**2′** are difficult to separate by HPLC (see Figure S13 in Supplementary Materials for radio HPLC examples). Moreover, [^18^F]**2′** will be able to react in the next step of peptide coupling in the same way as [^18^F]**2**. Therefore, a compromise has to be found between maximizing the conversion into [^18^F]**2** and minimizing [^18^F]**2′** formation. In addition, results with LAH were not reproducible, and ratios observed between [^18^F]**2** and [^18^F]**2′** were not constant. Therefore, other reducing agents were explored. Borane complexes are also standard reagents for nitrile reduction [43] which have been used for the reduction of [^18^F]fluorobenzonitrile [39]. In our case, reduction of [^18^F]**1** at either room temperature or 65 °C resulted in only 5% of [^18^F]**2** and mostly degradation of the starting material (Table 2, entries 3 and 4, respectively). Reduction of nitrile moieties by hydrogenation in the presence of palladium on charcoal has also been extensively described in the literature [44], although it has never been used for radiochemical reactions, due to the heterogeneity of the medium. In our case, hydrogenation of compound [^18^F]**1** in methanol at room temperature only resulted in the formation of compound [^18^F]**2′** with 30% conversion (Table 2, entry 5). Indeed, palladium-catalyzed hydrodefluorination has been reported in the case of poly-fluoro arenes [45]. Considering the sensitivity of [^18^F]**1** to defluorination and the significant proportion of side products formed using standard reducing agents, milder conditions were explored. Werkmeister et al. have described an original method to reduce nitrile by hydrogenation under mild conditions using [RuCl_2_(pCymene)_2_]_2_ and 3-bis(diphenylphosphino)propane (DPPP) as the catalytic system and isopropanol as the hydrogen donor [46]. Under those conditions, no side reactions were observed, but [^18^F]**2** was obtained with only 10% conversion (Table 2, entry 6). The in situ generation of metal boride using NaBH_4_ in the presence of transition metal (Co, Ni) and water is an efficient reducing agent to promote nitrile reduction [47]. This catalytic system has already been described for the reduction of [^18^F]fluorobenzonitrile to [^18^F]fluorobenzylamine [40,48,49]. Using excess amounts of NaBH_4_ and hexahydrated nickel chloride in methanol, [^18^F]**2** was obtained with 42% conversion, together with 22% of side products, mainly corresponding to the defluorinated compound [^18^F]**2′** (Table 2, entry 7). Way and Wuest described a supported version of this nitrile reduction using a borohydride exchange resin (BER) for the automated radiosynthesis of [^18^F]fluorobenzylamine [48]. In our hands, almost no reaction was observed (Table 2, entry 8).

Clearly the homogeneous nitrile reduction using NaBH_4_ and hexahydrated NiCl_2_ was the best compromise to achieve a moderate conversion into the desired benzylamine [^18^F]**2** while minimizing the formation of side products. In order to further improve the conversion into [^18^F]**2,** different parameters (quantity of reagents, solvent, time, and temperature) were studied (Table 3). THF was also explored to realize this reduction, but conversion into [^18^F]**2** was always lower than in methanol (data not shown). Decreasing the quantity of NaBH_4_ resulted in a total conversion of the starting material [^18^F]**1** but significantly increased the proportion of side products compared to the desired compound [^18^F]**2** (Table 3, entry 2). Increasing the time of reaction to 10 min (Table 3, entry 3) or the temperature to 50 °C (Table 3, entry 4) did not significantly increase the conversion into [^18^F]**2**. Additional water (100 µL) was introduced to the reaction to promote the formation of the Ni_2_B reducing agent and improve the homogeneity of the reaction (Table 3, entry 5). In those conditions, the conversion of [^18^F]**2** rose to 45%, with 55% of side products formed. To further improve the conversion of [^18^F]**2**, the influence of the quantity of NiCl_2_·6H_2_O was studied. Increasing the quantity to 20 µmol (Table 3, entry 6) or 40 µmol (Table 3, entry 7) did not result in significant improvement, whereas using 10 µmol of NiCl_2_·6H_2_O (Table 3, entry 8) afforded a conversion of 72% of [^18^F]**2** with only 28% of side products. In those conditions, increasing the reaction time to 10 min (Table 3, entry 9) did not yield any significant improvement of the conversion rate, whereas increasing the temperature to 50 °C (Table 3, entry 10) resulted in an increase of side product formation. Finally, the conversion of [^18^F]**2** dropped to 46% when increasing the quantity of NaBH_4_ while using 100 µmol of NiCl_2_·6H_2_O and additional water with unreacted [^18^F]**1** remaining (Table 3, entry 11). Overall, the best compromise to optimize the conversion into [^18^F]**2** (72%) while minimizing the formation of deleterious side products (28%) for the next reaction step was found when using a rather low quantity of NaBH_4_ (40 µmol) and a large excess of hexahydrated nickel chloride with additional water (Table 3, entry 8).

#### 2.2.3. Peptide Coupling Optimization

**Scheme 4:**
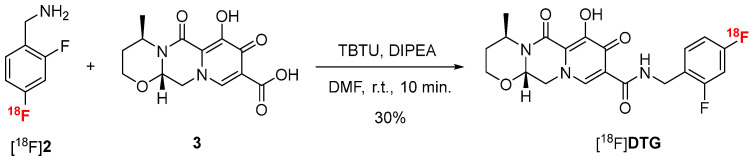
Peptide coupling reaction between [^18^F]**2** and compound **3** to afford [^18^F]DTG.

Inspired by the recent work of Dietz and colleagues [38], [^18^F]DTG was finally obtained in a last step of peptide coupling between [^18^F]**2** and carboxylic acid **3** using 2-(1H-benzotriazole-1-yl)-1,1,3,3-tetramethylaminium tetrafluoroborate (TBTU) as coupling agent and diisopropylethylamine (DIPEA) in DMF (Scheme 4). Compound **3** was synthesized in one-step from compound GSK3210346A [50] (see Scheme S3 in Supplementary Materials). In those conditions, [^18^F]DTG was obtained in 30% conversion after 10 min of reaction at room temperature. Increasing the temperature or the reaction time did not result in any significant improvement of the conversion.

### 2.3. Automated Radiosynthesis of [^18^F]DTG

**Figure 1:**
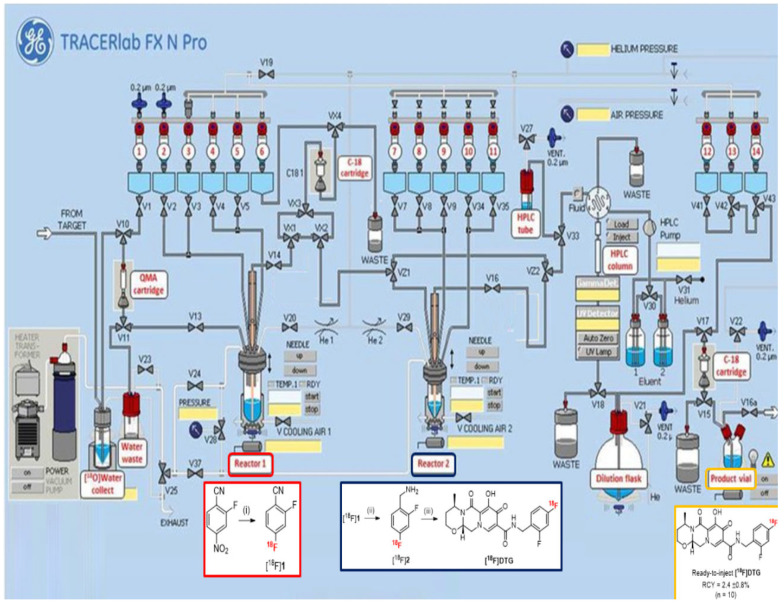
Automated radiosynthesis of [^18^F]DTG on a TRACERlab^®^ FX N Pro module. Radiofluorination (red square) takes place in reactor 1, whereas the nitrile reduction and peptide coupling reactions (blue square) take place in reactor 2. Ready-to-inject [^18^F]DTG (orange square) is obtained after semi-preparative HPLC purification and SPE formulation. Reagents and conditions: (i) K [^18^F]F, K_222_, K_2_CO_3_, DMSO, 95 °C, 5 min; (ii) NaBH_4_ 2M (20 µL), NiCl_2_·6H_2_O (25 mg), MeOH, H_2_O (100 µL), r.t., 5 min; (iii) **3**, TBTU, DIPEA, DMF, r.t., 10 min.

For in vivo PET imaging applications, the radiosynthesis of [^18^F]DTG has to be automated for reproducibility and radioprotection reasons. With optimized conditions for each step in hand, the automation was carried out using a TRACERlab^®^ FX N Pro (GE Healthcare, Chicago, IL, USA) module (Figure 1). The presence of two separated reactors on the module was mandatory, as the reaction conditions, the solvents in particular, between the fluorination and the reduction steps are not compatible. Moreover, unreacted fluoride ions could promote the decomposition of sodium borohydride [51]. In contrast, the reduction and the peptide coupling reaction could be realized in one pot in the second reactor. After using a standard procedure for the preparation of the K [^18^F]F/K_222_ complex with azeotropic drying, the radiofluorination step was realized in reactor 1 following the optimized conditions depicted above. In order to remove unreacted fluoride ions before the reduction step, the crude mixture from radiofluorination was pre-purified by solid-phase extraction (SPE) on a C18 cartridge. This step also achieved a solvent exchange from DMSO to methanol for the reduction step. The crude [^18^F]**1** was eluted with methanol from the C18 cartridge to the reactor 2 to avoid any contamination for the reduction step from possible residual DMSO or fluoride ions in reactor 1. To realize the reduction step, the sodium borohydride solution in methanol and the nickel chloride hexahydrate solution in water were added from separate vials into the reactor 2. Mixing the two solutions beforehand resulted in the degradation of the formed Ni_2_B reducing agent and a poor conversion into [^18^F]**2**. The coupling reaction was then performed in one pot by adding a mixture of compound **3**, TBTU, and DIPEA in DMF directly into reactor 2 at the end of the reduction step. At the end of the radiosynthesis, purification of the crude reaction mixture was realized by semi-preparative HPLC on a Symmetry C18 column (Waters) using a mixture of acetonitrile, water, and trifluoroacetic acid (TFA) as eluent. [^18^F]DTG was isolated with a retention time of about 17 min (see Figure S17 in Supplementary Materials). Final formulation by SPE on a C18 cartridge afforded ready-to-inject [^18^F]DTG in 95 min and 5.1 ± 0.8% (*n* = 10) decay-corrected radiochemical yield. This yield is comparable to the similar radiosynthesis of [^18^F]Raltegravir (4.61 ± 0.3% within 135 min) described by Blecha et al. [37]. The quantity of [^18^F]DTG produced was in the range 500–700 MBq, consistent with administration to humans (typically in the range 200–400 MBq).

### 2.4. Quality Control

**Figure 2:**
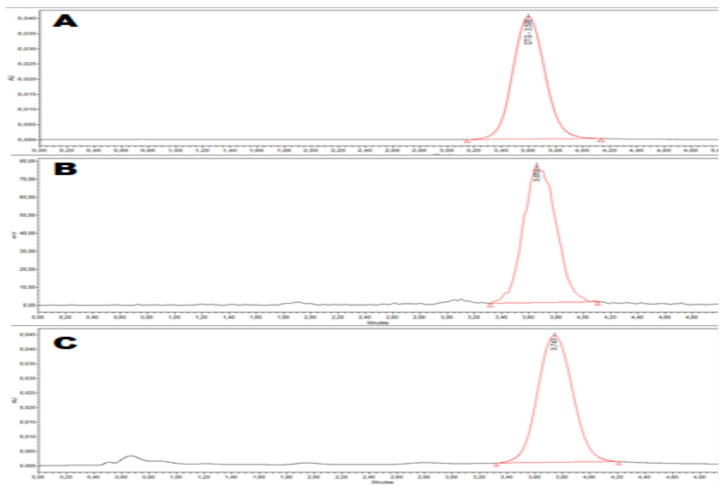
Quality control of [^18^F]DTG by analytical HPLC. (**A**) UV (258 nm) chromatogram of the reference compound DTG; (**B**) gamma chromatogram of the radiotracer [^18^F]DTG; (**C**) UV (258 nm) chromatogram of the radiotracer [^18^F]DTG.

Quality control was performed on [^18^F]DTG by analytical HPLC with both UV (258 nm) and gamma detection to confirm the identity of the radiotracer and its chemical and radiochemical purity and to assess the molar activity (MA) of the radiotracer (Figure 2). The identity of the radiotracer was confirmed by the retention time of [^18^F]DTG (t_R_ = 3.74 min, Figure 2C), which was within the range t_R_^ref^ ± 10% compared to the retention time of the DTG reference (t_R_^ref^ = 3.60 min., Figure 2A). [^18^F]DTG was obtained with both a radiochemical and chemical purity above 99% (Figure 2B and C respectively). [^18^F]DTG was obtained with a MA of 83 ± 18 GBq/µmol (*n* = 10) calculated with the help of a calibration curve (see Figure S18 in Supplementary Materials). In summary, the quality control of [^18^F]DTG demonstrated that the radiotracer was suitable for in vivo injection to perform PET imaging. Pending additional tests, [^18^F]DTG would be suitable for human injection according to guidance from the European Pharmacopeia [52].

### 2.5. PET Imaging

**Figure 3:**
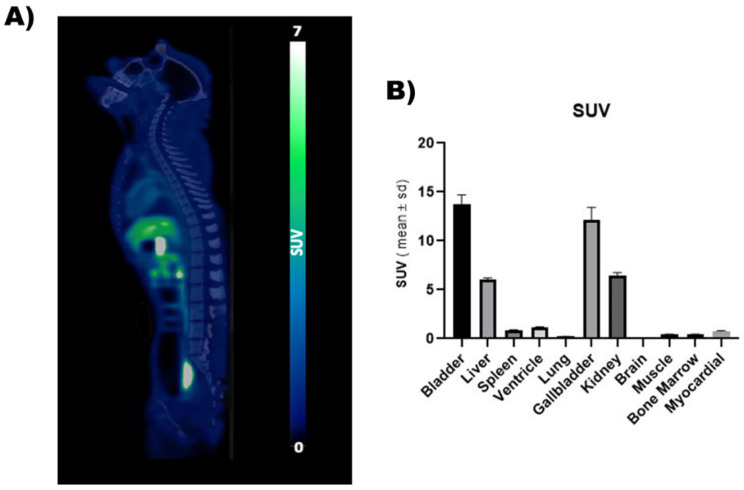
PET imaging of a non-human primate using [^18^F]DTG. (**A**) Representative whole-body [^18^F]DTG PET-CT image obtained in a healthy macaque. PET signal is expressed in SUV units. (**B**) SUV (calculated as the mean value at the equilibrium between 150 and 180 min) in the organs of interest.

Feasibility of in vivo PET imaging using [^18^F]DTG was first assessed in a non-human primate. PET images unveiled the whole-body distribution of [^18^F]DTG (Figure 3). Highest PET signal was observed in the liver (SUV = 6.06 ± 0.14), kidneys (SUV = 6.45 ± 0.31), gallbladder (SUV = 12.16 ± 1.26), and urinary bladder (SUV = 13.73 ± 0.97), consistent with the known elimination routes of DTG [53]. PET signal in the brain was strikingly low (SUV = 0.04 ± 0.001) compared with peripheral organs, suggesting limited brain penetration of [^18^F]DTG. Minimally invasive [^18^F]DTG PET, therefore, sheds light on the elimination of DTG and its distribution to tissues. Interestingly, it suggests a minimal distribution to the brain which could correspond to a pharmacological sanctuary. Previous studies suggest a low brain distribution of [^18^F]DTG in mice [54]. Further experiments with dynamic brain PET imaging and kinetic modelling will however be necessary to correctly estimate the brain distribution of [^18^F]DTG in macaques.

## 3. Materials and Methods

### 3.1. Chemistry

Chemicals were purchased from Aldrich (Saint-Quentin-Fallavier, France) and used as received. 2-Fluoro-4-nitrobenzonitrile was purchased from Fluorochem. Dolutegravir and compound GSK3210346A were kindly provided by GSK. Reactions were monitored by thin layer chromatography (TLC) on aluminum pre-coated plates of silica gel 60F_254_ (VWR, Rosny-sous-bois, France). The compounds were localized at 254 nm using a UV lamp. ^1^H and ^13^C NMR spectra were recorded on a Bruker Advance 400 MHz apparatus using DMF-*d*_4_ as solvent. The chemical shifts (δ) are reported in ppm (s, d, t, q, and b for singlet, doublet, triplet, quadruplet, and broad signal, respectively) and referenced with the solvent residual chemical shift. Ultra-performance liquid chromatography-mass spectroscopy (UPLC-MS) was realized on an Ultimate 3000 (Thermo Scientific, Waltham, MA, USA) device equipped with an Acquity BEH 2.1 × 50 mm, 1.7 µm column (Waters, Milford, MA, USA). A gradient of water with 0.1% of formic acid and acetonitrile with 0.1% of formic acid (3% of CH_3_CN/HCHO for 2 min, then rising to 100% during 7 min, then decreasing to 3% during 1 min, then keeping 3% for 2 min) at a flow rate of 0.3 mL/min was used. Mass spectroscopy was performed with a Linear Trap Quadripole Orbitrap Velos (Thermo Scientific, Waltham, MA, USA) equipped with an electron spray ionization (ESI) chamber. Spectra were recorded between 100 and 1000 *m*/*z*. High-resolution mass spectrometry (HRMS) analyses were performed by the Small Molecule Mass Spectrometry platform of ICOA, (Orléans, France) by electrospray with positive (ESI+) ionization mode.

**(*4R*,*12aS*)-7-hydroxy-4-methyl-6,8-dioxo-3,4,6,8,12,12a-hexahydro-2H-pyrido[1′,2′:4,5]pyrazino[2,1-b][1,3]oxazine-9-carboxylic acid** (**3**). To a solution of GSK3210346A (500 mg, 1.0 equiv.) in DMF (20 mL) was added lithium chloride (690 mg, 10.0 equiv.), and the mixture was heated for 5 h at 120 °C. Upon cooling to room temperature, an aqueous solution of HCl (1 M, 10 mL) was added to reach a pH of 1. The aqueous phase was extracted with ethyl acetate (2 × 10 mL), and the combined organic phases were washed with a saturated solution of sodium chloride (2 × 10 mL), dried over sodium sulfate, and the solvent was evaporated. The residue was dried under vacuum to afford compound 3 (360 mg, 76%) as a white powder. ^1^H-NMR (DMF-*d*_4_, 400 MHz) δ 15.39 (s, 1H), 12.94 (bs, 1H), 8.70 (s, 1H), 5.68 (q, J = 4 Hz, 1H), 4.92–4.84 (b, 2H), 4.57 (dd, J^2^ = 14 Hz, J^3^ = 6 Hz, 1H), 4.17 (td, J^2^ = 12 Hz, J^3^ = 2 Hz, 1H), 4.00 (dq, J^2^ = 12 Hz, J^3^ = 2 Hz, 1H), 2.22–2.12 (b, 1H), 1.69–1.65 (b, 1H), 1.43 (d, J = 7 Hz, 3H) ppm. ^13^C-NMR (DMF-*d*_4_, 100 MHz) δ 172.9, 165.6, 162.0, 154.6, 141.2, 118.6, 113.6, 76.6, 62.4, 52.2, 45.3, 29.5, 14.9 ppm. HR-ESI(+)-MS *m*/*z* calcd for C_13_H_15_N_2_O_6_: 295.0925 [M + H]^+^, found 295.0923. NMR and mass spectra are presented in Suppporting Information (Figure S14, S15 and S16 respectively).

### 3.2. Radiochemistry

All reactions were carried out using a TRACERlab^®^ FX N Pro module (GE Healthcare).

No carrier-added [^18^F]fluoride ion was produced via the ^18^O(p, n)^18^F nuclear reaction by irradiation of a 2 mL [^18^O]water (>97% enriched, Rotem) target with an IBA Cyclone-18/9 (IBA) cyclotron.

Analytical HPLC was performed using a 717_plus_ Autosampler system, a 1525 binary pump, a 2996 photodiode array detector (Waters, Milford, MA, USA), and a Flowstar LB 513 (Berthold, Thoiry, France) gamma detector. The system was operated with the Empower 3 (Waters, Milford, MA, USA) software. HPLC were realized on a reverse phase analytical Symmetry^®^ C18 (150 × 3.9 mm, 5 μm, Waters) column using a mixture of H_2_O/CH_3_CN/PicB7^®^ as eluent. The chemical identification of the peak was assessed by comparing the retention time of the radiotracer with the retention time of the non-radioactive reference (t_R_^ref^). For acceptance, the retention time must be within the t_R_^ref^ ± 10% range. Radiochemical and chemical purities were calculated as the ratio of the AUC of the radiotracer peak over the sum of the AUCs of all other peaks on gamma and UV chromatograms, respectively. The conversion of the reaction was calculated as the ratio of the decay-corrected activity of the radiotracer at the end of the synthesis over the activity of the starting material, both measured in an ionization chamber (Capintec^®^, Berthold, Thoiry, France).

**2-Fluoro-4-([^18^F]fluoro)benzonitrile ([^18^F]1).** [^18^F]F^−^ (2–3 GBq) was trapped on an ion exchange resin QMA light (Waters, Milford, MA, USA) and eluted with a solution of K_2_CO_3_ (2 mg) in a mixture of CH_3_CN (800 µL) and H_2_O (200 µL) containing Kryptofix-222 (12–15 mg). The resulting complex was dried upon heating at 60 °C for 7 min under vacuum and a stream of helium followed by heating at 120 °C for 5 min under vacuum only. Upon cooling to 70 °C, a solution of 2-fluoro-4-nitrobenzonitrile (5 mg) in the solvent (CH_3_CN or DMSO, 700 µL) was added and the mixture was heated at 95 °C for 5, 10, or 15 min. Upon cooling to room temperature, the mixture was diluted with CH_3_CN (2 mL) and analyzed by TLC on aluminum pre-coated plates of silica gel 60F_254_ (VWR, Rosny-sous-bois, France).) using a mixture of dichloromethane/methanol 9/1 *v*/*v* as eluent. TLC plates were analyzed with a Mini-scan TLC imaging scanner equipped with a FC-3600 beta probe (Bioscan Inc., Washington, DC, WA, USA). TLC chromatograms were recorded using the Chromeleon^TM^ (ThermoFischer, Waltham, MA, USA) software.

**2-Fluoro-4-([^18^F]fluoro)benzylamine ([^18^F]2).** To a solution of [^18^F]**1** (tracer quantity) in the appropriate solvent (1 mL) were added the reduction agents, and the reaction took place under the conditions depicted in Table 2 and Table 3. The reaction was quenched using a mixture of CH_3_CN/H_2_O 1/1 *v*/*v* (1 mL), and the crude was analyzed by HPLC according to the general procedure. A mixture of H_2_O/CH_3_CN/Et_3_N (80/20/0.1 *v*/*v*/*v*, 2 mL/min) was used as eluent, and UV detection was performed at 254 nm.

**[^18^F]Dolutegravir ([^18^F]DTG).** To a solution of [^18^F]**2** (tracer quantity) in DMF (500 µL) were added compound **3** (12 mg), TBTU (13 mg), and DIPEA (15 µL). The reaction was stirred at room temperature for 10 min and quenched with a mixture of CH_3_CN/H_2_O 1/1 *v*/*v* (1 mL). The crude was analyzed by HPLC according to the general procedure. A mixture of H_2_O/CH_3_CN/PicB7^®^ (70/30/0.2, 2 mL/min) was used as eluent, and UV detection was performed at 258 nm.

**Fully automated radiosynthesis of [^18^F]Dolutegravir.** [^18^F]F^−^ (20–30 GBq) was trapped on an ion exchange resin QMA light (Waters) and eluted in reactor 1 with a solution of K_2_CO_3_ (2 mg) in a mixture of CH_3_CN (800 µL) and H_2_O (200 µL) containing Kryptofix-222 (12–15 mg). The resulting complex was dried upon heating at 60 °C for 7 min under vacuum and a stream of helium followed by heating at 120 °C for 5 min under vacuum only. Upon cooling to 70 °C, a solution of 2-fluoro-4-nitrobenzonitrile (5 mg) in DMSO (700 µL) was added, and the mixture was heated at 95 °C for 5 min. After cooling to room temperature, the mixture was diluted with water (10 mL) and passed through a C18 Sep Pak^®^ cartridge (Waters). The cartridge was rinsed with water (5 mL) and eluted with methanol (1.5 mL) to reactor 2. A solution of NaBH_4_ 2M (20 µL) in THF (100µL) was added, followed by the addition of a solution of NiCl_2_·6H_2_O (25 mg) in water (100 µL). The mixture was stirred at room temperature for 5 min. A solution of compound **3** (12 mg), TBTU (13 mg), and DIPEA (15 µL) in DMF (500 µL) was added, and the mixture was stirred at room temperature for 10 min. The reaction was diluted with a mixture of CH_3_CN/H_2_O/TFA 30/70/0.1 *v*/*v*/*v* (1.5 mL), and the crude was purified by reverse phase semi-preparative HPLC (Waters Symmetry^®^ C18 7.8 × 300 mm, 7 μm) with a 515 HPLC Pump (Waters, Milford, MA, USA) using a mixture of H_2_O/CH_3_CN/TFA (70/30/0.1 *v*/*v*/*v*, 5 mL/min) as eluent. UV detection (K2501, Knauer, Berlin, Germany) was performed at 254 nm. The purified compound was diluted with water (20 mL) and passed through a Sep-Pak^®^ C18 cartridge (Waters, Milford, MA, USA). The cartridge was rinsed with water (10 mL) and eluted with ethanol (2 mL), and the final compound was diluted with saline (0.9% *w*/*v*, 8 mL). Ready-to-inject [^18^F]DTG (0.5–0.7 GBq) was obtained in 5.1 ± 0.8% (*n* = 10) non-decay corrected radiochemical yield within 95 min.

**Quality control.** Quality control was performed by reverse phase analytical HPLC according to the general procedure described above. A mixture of H_2_O/CH_3_CN/PicB7^®^ (70/30/0.2 *v*/*v*/*v*, 2 mL/min) was used as eluent, and UV detection was performed at 258 nm. Molar activity was calculated as the ratio of the activity of the collected peak of [^18^F]DTG measured in an ionization chamber (Capintec^®^, Berthold, Thoiry, France) over the molar quantity of DTG determined using a calibration curve (see Figure S18 in Supplementary Materials). Molar activity is calculated as the mean value of three consecutive runs.

### 3.3. PET Imaging

Whole-body distribution of [^18^F]DTG was assessed using whole-body (3-bed step) PET acquisition in a healthy male macaque (Macaca fascicularis, 8.35 kg, 10 years). Animal was i.v. injected with 181 MBq of ^18^F-DTG followed by PET acquisition performed using a Siemens Biograph PET-CT scanner (Siemens Healthineers, Knoxville, TN, USA) as previously described [55]. Mean PET signal acquired from 150 to 180 min after injection of [^18^F]DTG was normalized by injected dose of [^18^F]DTG and animal weight (standardized uptake value, SUV). Representative PET image was co-registered to the anatomical CT scan.

## 4. Conclusions

We have developed the first fluorine-18 isotopic radiolabeling of the antiretroviral drug dolutegravir. The three steps of the radiosynthesis, i.e., radiofluorination, reduction, and peptide coupling reactions, have been optimized to maximize the quantity of [^18^F]DTG produced. The whole process has been automated for reproducibility and radioprotection purposes to ensure the feasibility of preclinical PET imaging studies. For that matter, quality control has been set up, and 500–700 MBq of ready-to-inject [^18^F]DTG was produced, a quantity compatible with injection into primates or humans. As a proof of concept, the first in vivo injection of [^18^F]DTG was carried out in a macaque, and PET imaging was performed to observe the biodistribution of the tracer. [^18^F]DTG is currently being used in PET studies with healthy and simian immunodeficiency virus (SIV)-positive non-human primates to explore the distribution of cART at remaining SIV sanctuaries and to understand their resistance.

## Figures and Tables

**Table 1:** Reaction conditions for the radiofluorination of 2-fluoro-4-nitrobenzonitrile.

Entry ^1^	Solvent	Reaction Time	Conversion in [^18^F]1 ^2^
1	CH_3_CN	5 min	96%
2	CH_3_CN	10 min	97%
3	CH_3_CN	15 min	97%
4	DMSO	5 min	90%

^1^ Experiments were realized in duplicates. ^2^ Conversion rate was calculated as the ratio of formed [^18^F]**1** over the total radioactivity measured by TLC. TLC are presented in Figure S12 in Supplementary Materials.

**Table 2:** Different reducing agents explored for the nitrile reduction of compound [^18^F]**1** into [^18^F]**2**.

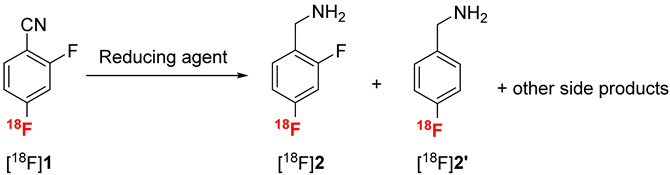				
Entry ^1^	Reducing Agents	Reaction Conditions	[^18^F]2 Conversion ^2^	Side Products Conversion ^2^
1	LAH (100 µmol)	THF, r.t., 5min	15%	85%
2	LAH (100 µmol)	THF, 50 °C, 5min	50%	50%
3	BH_3_.THF (200 µmol)	THF, r.t., 15 min	5%	91%
4	BH_3_.THF (200 µmol)	THF, 65 °C, 5 min	5%	91%
5	H_2_ (1 atm)	Pd/C (150 µg), MeOH, r.t., 5 min	0%	30%
6	[RuCl_2_(*p*-cymene)_2_] (1.5 µmol)	DPPP, NaOH 3M iPrOH, 100 °C, 15 min	10%	-
**7**	**NaBH_4_ (400 µmol),** **NiCl_2_ 6H_2_O (4 µmol)**	**MeOH, r.t., 5 min**	**42%**	**22%**
8	BER (500 mg), NiCl_2_ 6H_2_O (20 mg)	MeOH	<5%	-

^1^ Experiments were performed at least in duplicate. ^2^ Conversion rates of [^18^F]**2** and side products were measured by radio-HPLC as the ratio of formed [^18^F]**2** or side products, respectively, over the total radioactivity. Best conditions were highlighted in red.

**Table 3:** Optimization of the nitrile reduction using NaBH_4_ (2M) and NiCl_2_·6H_2_O as reducing agents.

Entry ^1^	NaBH_4_ (µmol)	NiCl_2_·6H_2_O (µmol)	H_2_O (µL)	T (°C)	Time (min)	[^18^F]2 Conversion ^2^	Side Products Conversion ^3^
1	400	4	-	r.t.	5	42%	22%
2	40	4	-	r.t.	5	20%	80%
3	40	4	-	r.t.	10	23%	77%
4	40	4	-	50 °C	5	26%	74%
5	40	4	100	r.t.	5	45%	55%
6	40	20	100	r.t.	5	47%	53%
7	40	40	100	r.t.	5	41%	59%
8	**40**	**100**	**100**	**r.t.**	**5**	**72%**	**28%**
9	40	100	100	r.t.	10	69%	31%
10	40	100	100	50 °C	5	65%	35%
11	200	100	100	r.t.	5	46%	40%

^1^ Experiments were realized in duplicate. ^2^ The conversion rate was measured by radio-HPLC as the ratio of formed [^18^F]**2** over the total radioactivity. The conversion is the mean value of two experiments. ^3^ The side products represent the combination of [^18^F]**2′** and other unidentified compounds formed during the reduction process. Best conditions were highlighted in red.

## Data Availability

Data is contained within article and Supplementary Materials.

## Supplementary Materials

The following supporting information can be downloaded at: https://www.mdpi.com/article/10.3390/ph15050587/s1, Scheme S1: Synthesis of late-stage labelling precursors of DTG.; Scheme S2: Radiolabelling of the tributyltin precursor s5 according to the conditions of the literature; Figure S1: ^1^H NMR of compound **s1**; Figure S2: ^13^C NMR of compound **s1**; Figure S3: HRMS analysis of compound **s1**; Figure S4: ^1^H NMR of compound **s2**; Figure S5: ^13^C NMR of compound **s2**; Figure S6: HRMS analysis of compound **s2**; Figure S7: LC-MS^−^ analysis of crude compound **s3**; Figure S8: LC-MS^+^ analysis of crude compound **s4**; Figure S9: ^1^H NMR of compound **s5**; Figure S10: ^13^C NMR of compound **s5**; Figure S11: HRMS analysis of compound **s5**; Figure S12: TLC analysis of the radiofluorination step; Figure S13: HPLC analysis of a mixture of compounds **1**, **2** and **2′**; Scheme S3: Synthesis of compound **3** from GSK3210346A; Figure S14: ^1^H NMR of compound **3**; Figure S15: ^13^C NMR of compound **3**; Figure S16: HRMS analysis of compound **3**; Figure S17: Semi-preparative HPLC purification of [^18^F]DTG; Figure S18: Calibration curve of DTG for molar activity calculation.
