# Supplementary material for: Isotopic Radiolabeling of the Antiretroviral Drug [18F]Dolutegravir for Pharmacokinetic PET Imaging
Source: Pharmaceuticals (Basel). 2022 May 10;15(5):587. doi: 10.3390/ph15050587 (PMC9143889; doi:10.3390/ph15050587)
Supplement: Supplementary file 1 [file pharmaceuticals-15-00587-s001.zip › pharmaceuticals-1649366-supplementary.pdf]

Supporting information

Isotopic radiolabelling of the antiretroviral drug [^{18}F]Dolutegravir for pharmacokinetic PET imaging

Marion Tisseraud[1], Sébastien Goutal[1], Thomas Bonasera[2], Maud Goislard[1], Delphine Desjardins[3], Roger Legrand[3], Chris M. Parry[4], Nicolas Tournier[1], Bertrand Kuhnast[1] and Fabien Caillé[1,*]

[1]Université Paris-Saclay, Inserm, CNRS, CEA, Laboratoire d'Imagerie Biomédicale Multimodale Paris-Saclay (BioMaps), 91401 Orsay

[2]GSK Medicines Research Centre, Gunnels Wood Road, Stevenage, Hertfordshire, SG1 2NY, United Kingdom

[3]Université Paris-Saclay, Inserm, CEA, Center for Immunology of Viral, Auto-immune, Hematological and Bacterial diseases (IMVA-HB/IDMIT), Fontenay-aux-Roses, France.

[4]ViiV Healthcare, 980 Great West Road, London, TW8 9GS, United Kingdom

Synthesis of late-stage labelling precursors of DTG

The late-stage labelling precursors of DTG were synthesized in two steps from **GSK3210346A**. Peptide couplings with 2-fluoro-4-halidebenzylamines afforded either the iodinated compound **s1** or the brominated compound **s2** in 64% and 57% yields respectively (Scheme S1). The iodonium ylide precursor **s3** was synthesized in 34 % yield from **s1** by oxidation of the iodine followed by reaction with Meldrum's acid. Compound **s3** could not be separated from the starting material and was not stable above 120°C. It was therefore considered not suitable for radiolabelling with fluorine-18. Boronic ester derivative **s4** was synthesized from compound **s2** by palladium catalysed coupling reactions and identified by LC/MS analysis but was too unstable to be isolated. The tributyltin precursor **s5** was also synthesized by palladium-catalyzed reaction from compound **s2** and isotaled with 40 % yield.

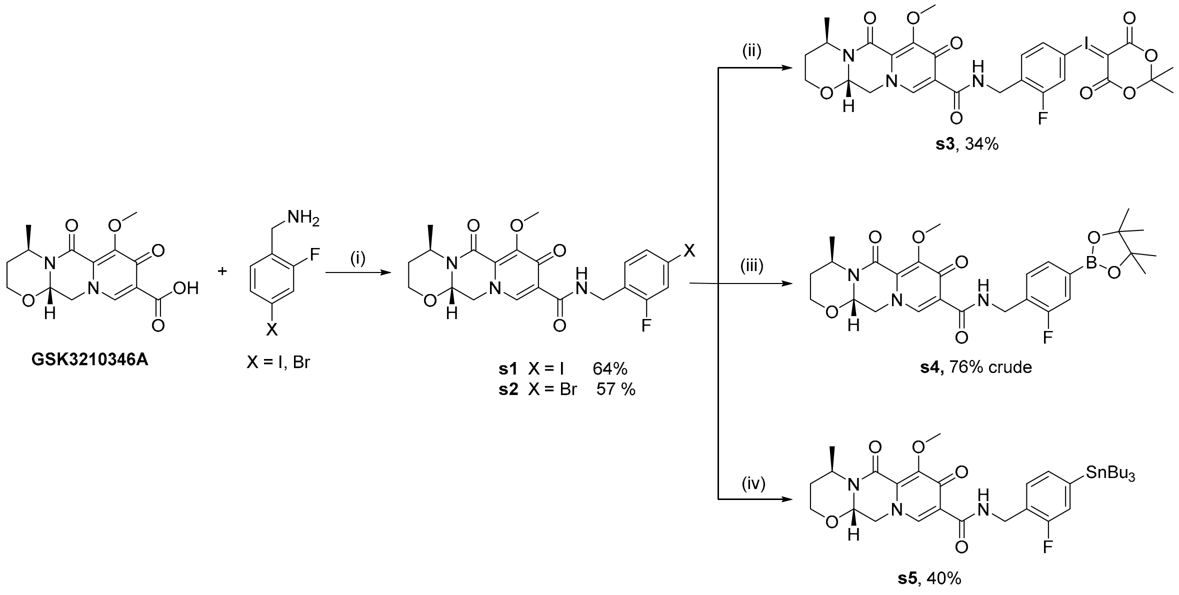

Scheme S1. Synthesis of late-stage labelling precursors of DTG. Reagents and conditions: (i) TBTU, DIPEA, DMF, r.t. 72h ; (ii) *m*CPBA, $CHCl_3$, r.t. 20h then Meldrum's acid, Na_2CO_{3aq} 10%, EtOH, r.t., 48h ; (iii) KOAc, $B_2(Pin)_2$, $PdCl_2(dppf)$, 1,4-dioxane, 80 °C, 20h ; (iv) $Sn_2(^nBu)_6$, $Pd(PPh_3)_4$, 1,4-dioxane, Et_3N, 110 °C, 17h.

Radiolabelling of compound **s5** was attempted by copper-catalyzed Chan-Lam like fluorination using the conditions described in the literature for trialkyltin precursors (Scheme S2).[1] Using dimethylacetamide (DMA) as solvent, different temperature ranging from 120 to 160 °C and different reaction time from 10 to 20 min. were explored, together with different loads of copper catalyst (5-15 mg for 4 mg of **s5**) but desired compound [^{18}F]**s6** could never been observed.

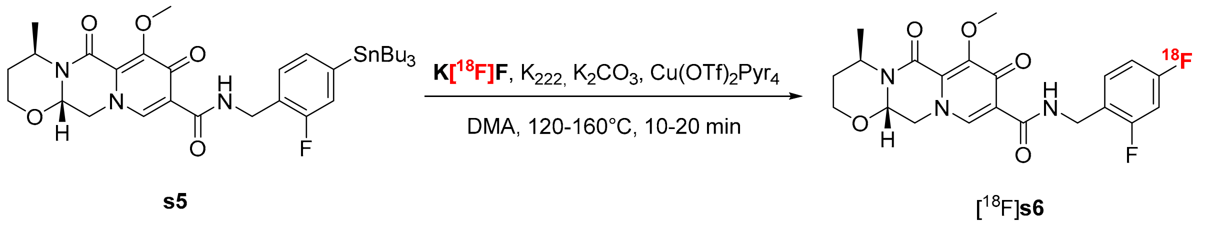

Scheme S2. Radiolabelling of the tributyltin precursor **s5** according to the conditions of the literature[1]

(4R,12aS)-*N*-(2-fluoro-4-iodobenzyl)-7-methoxy-4-methyl-6,8-dioxo-3,4,6,8,12,12a-hexahydro-2H-pyrido[1',2':4,5]pyrazino[2,1-b][1,3]oxazine-9-carboxamide (s1)

To a solution of compound GSK3210346A (1.0 g, 1.0 equiv.) in DMF (40 mL) were added (4-iodo-2-fluorophenyl)methanamine hydrochloride (1.1 g, 1.2 equiv.), TBTU (1.2 g, 1.2 equiv.) and DIPEA (660 µL, 1.2 equiv.). The solution was stirred for 72h at room temperature and water (50 mL) was added. The organic phase was extracted with ethyl acetate (3 x 20 mL) and the combined organic phases were washed with a solution of hydrochloric acid 1 M (2 x 20 mL), a saturated solution of sodium hydrogenocarbonate (2 x 20 mL), water (20 mL) and a saturated solution of sodium chloride (20 mL). The organic phase was dried with sodium sulfate and the solvent was evaporated. The residue was dried under vacuum to afford compound **s1** (1.1 g, 64 %) as a white powder. ^{1}H-NMR ($CDCl_3$, 400 MHz) δ 10.41 (t, J = 6 Hz , 1H), 8.41 (s, 1H), 7.41 (td, J^2 = 8 Hz, J^3 = 1 Hz, 2H), 7.11 (t, J = 8 Hz, 1H), 5.20 (q, J = 4 Hz, 1H), 5.05-4.98 (b, 1H), 4.60 (d, J = 6 Hz, 2H), 4.29 (dd, J^2 = 13 Hz, J^3 = 4 Hz, 1H), 4.13 (dd, J^2 = 13 Hz, J^3 = 6 Hz, 1H), 4.03 (s, 3H), 3.97 (dd, J^2 = 9 Hz, J^3 = 2 Hz, 2H), 2.23-2.13 (b, 1H), 1.53 (qd, J^2 = 14 Hz, J^3 = 2 Hz, 1H), 1.36 (d, J = 7 Hz, 3H) ppm. ^{13}C-NMR ($CDCl_3$, 100 MHz) δ 174.6, 164.0, 160.0 (d, J = 251 Hz), 155.7, 154.7, 142.2, 133.4 (d, J = 3 Hz), 131.0 (J = 4 Hz), 129.2, 125.5 (d, J = 15 Hz), 124.6 (d, J = 24 Hz), 118.9, 91.8 (d, J = 8 Hz), 76.1, 62.5, 61.2, 53.5, 44.6, 36.7 (d, J = 4 Hz), 29.4, 16.0 ppm. HR-ESI(+)-MS m/z calcd for $C_{21}H_{22}FIN_3O_5$: 542.0583 $[M+H]^+$, found 542.0583.

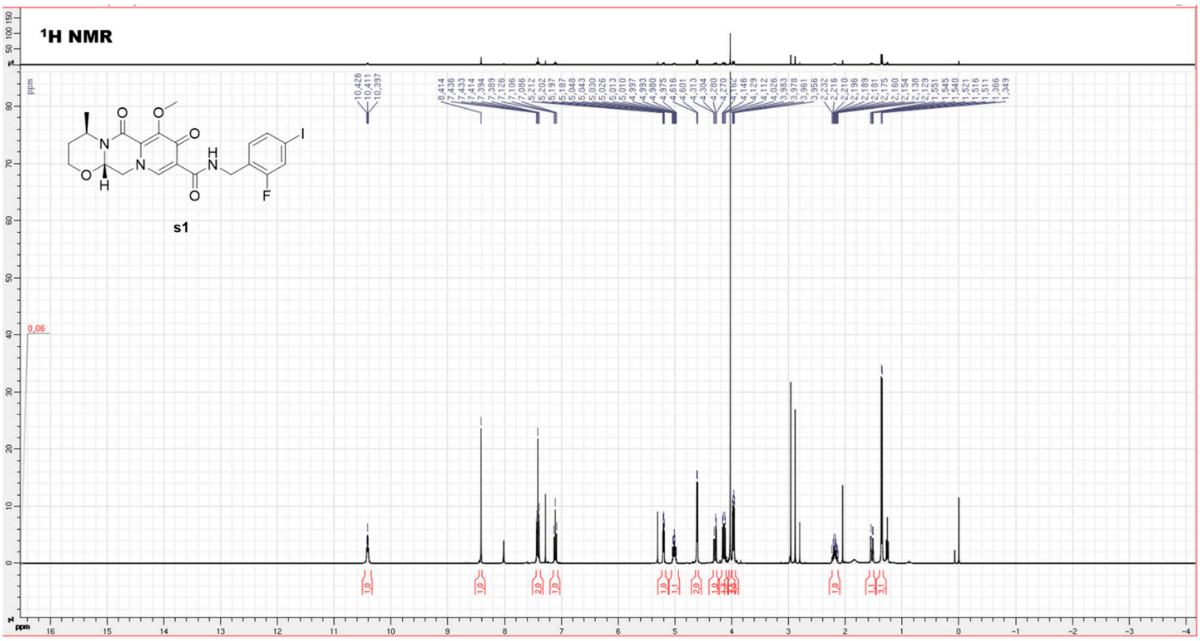

Figure S1. ^{1}H NMR of compound **s1**.

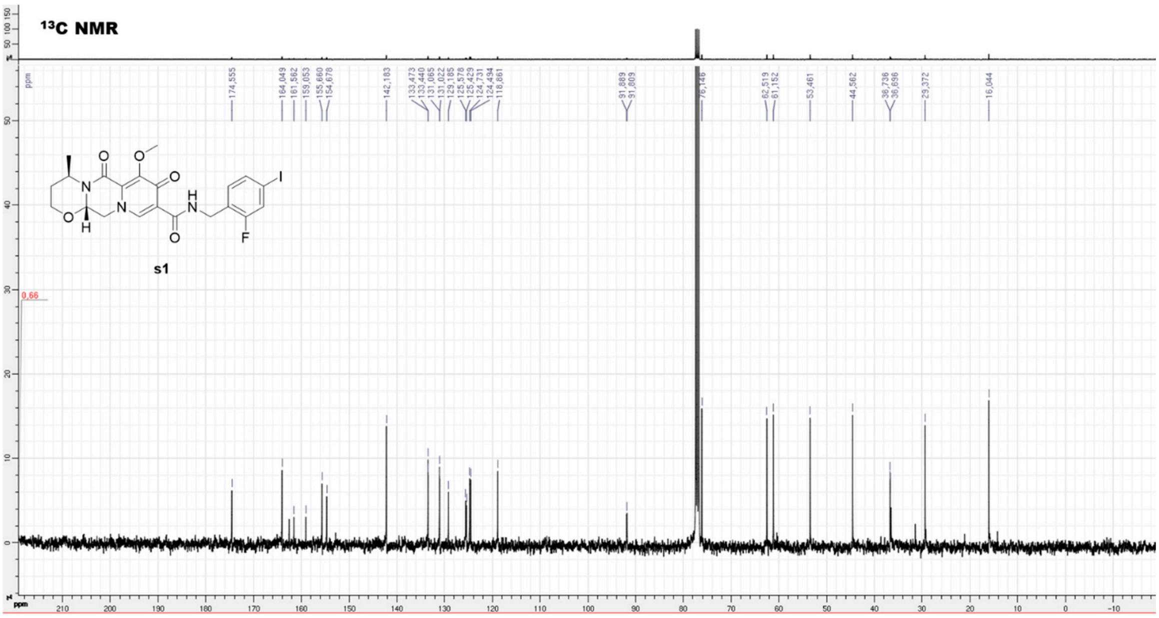

Figure S2. ^{13}C NMR of compound **s1**.

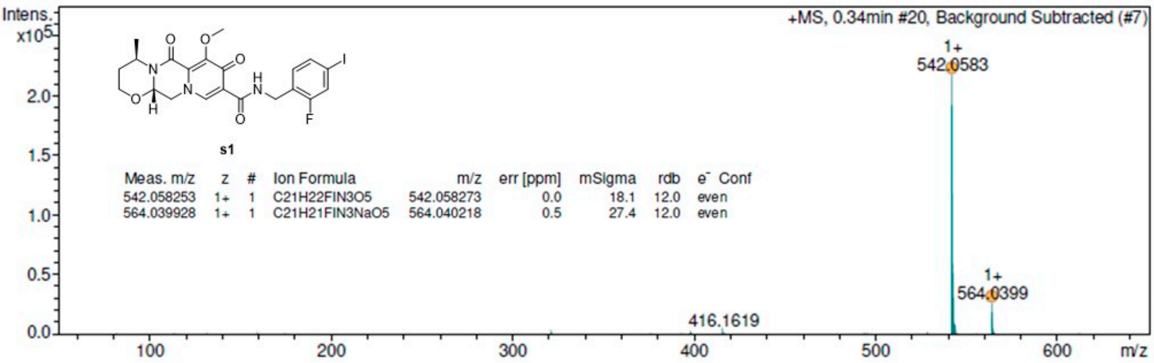

Figure S3. HRMS analysis of compound **s1**.

(4R,12aS)-*N*-(4-bromo-2-fluorobenzyl)-7-methoxy-4-methyl-6,8-dioxo-3,4,6,8,12,12a-hexahydro-2H-pyrido[1',2':4,5]pyrazino[2,1-b][1,3]oxazine-9-carboxamide (s2)

To a solution of compound GSK3210346A (1.0 g, 1.0 equiv.) in DMF (40 mL) were added (4-bromo-2-fluorophenyl)methanamine (790 mg, 1.2 equiv.), TBTU (1.2 g, 1.2 equiv.) and DIPEA (660 µL, 1.2 equiv.). The solution was stirred for 72h at room temperature and water (50 mL) was added. The organic phase was extracted with diethyl ether (3 x 20 mL) and the combined organic phases were washed with a solution of hydrochloric acid 1 M (2 x 20 mL), a saturated solution of sodium hydrogenocarbonate (2 x 20 mL), water (20 mL) and a saturated solution of

sodium chloride (20 mL). The organic phase was dried with sodium sulfate and the solvent was evaporated. The residue was dried under vacuum to afford compound **s2** (900 mg, 57 %) as a white powder. ^{1}H-NMR ($CDCl_3$, 400 MHz) δ 10.41 (t, J = 6 Hz , 1H), 8.42 (s, 1H), 7.26-7.21 (b, 3H), 5.20 (q, J = 4 Hz, 1H), 5.05-4.98 (b, 1H), 4.61 (d, J = 6 Hz, 2H), 4.29 (dd, J^2 = 14 Hz, J^3 = 4 Hz, 1H), 4.14 (dd, J^2 = 14 Hz, J^3 = 6 Hz, 1H), 4.03 (s, 3H), 3.97 (dd, J^2 = 9 Hz, J^3 = 2 Hz, 2H), 2.23-2.14 (b, 1H), 1.53 (qd, J^2 = 14 Hz, J^3 = 2 Hz, 1H), 1.35 (d, J = 7 Hz, 3H) ppm. ^{13}C-NMR ($CDCl_3$, 100 MHz) δ 174.6, 164.0, 160.0 (d, J = 250 Hz), 155.7, 154.7, 142.2, 130.7 (d, J = 5 Hz), 129.2, 127.4 (J = 3 Hz), 124.7 (d, J = 15 Hz), 121.1 (d, J = 9 Hz), 119.1, 118.9, 76.1, 62.5, 61.2, 53.5, 44.6, 36.7 (d, J = 4 Hz), 29.4, 16.0 ppm. HR-ESI(+)-MS m/z calcd for $C_{21}H_{22}FBrN_3O_5$: 494.0721 $[M+H]^+$, found 494.0719.

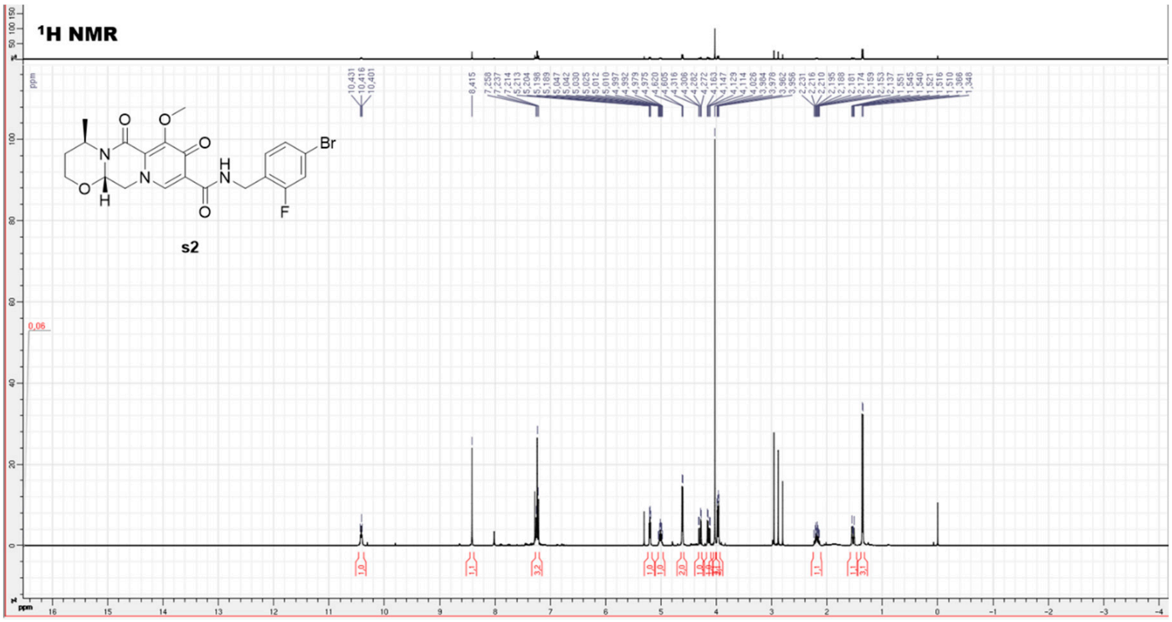

Figure S4. ^{1}H NMR of compound **s2**.

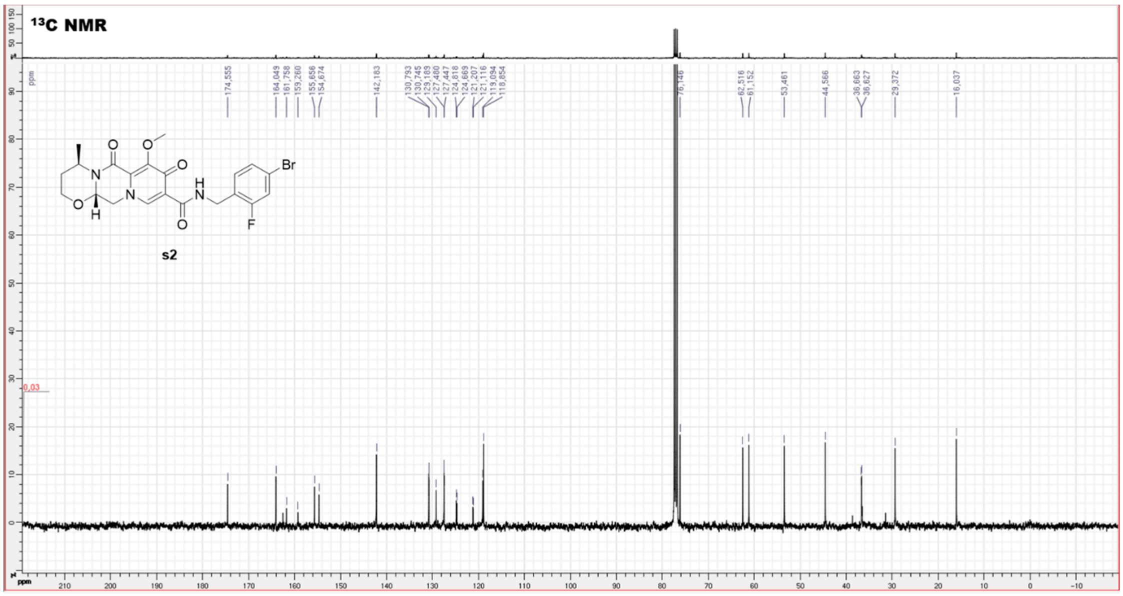

Figure S5. ^{13}C NMR of compound **s2**.

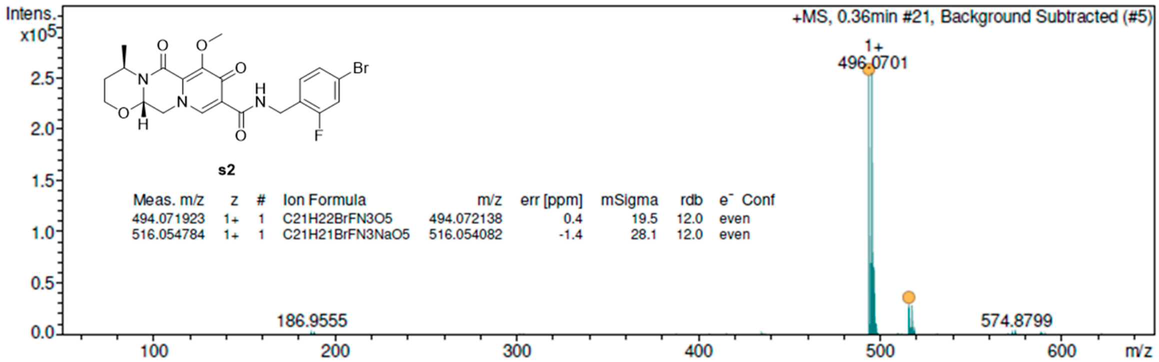

Figure S6. HRMS analysis of compound **s2**.

(4R,12aS)-*N*-(4-((2,2-dimethyl-4,6-dioxo-1,3-dioxan-5-ylidene)-l3-iodaneyl)-2-fluorobenzyl)-7-methoxy-4-methyl-6,8-dioxo-3,4,6,8,12,12a-hexahydro-2H-pyrido[1',2':4,5]pyrazino[2,1-b][1,3]oxazine-9-carboxamide (s3)

To a solution of **s1** (14 mg, 1.0 equiv.) in chloroform (1.5 mL) under argon was added m-CPBA (30 mg, 6.0 equiv.). The mixture was stirred at room temperature for 20h and the solvent was evaporated. The residue was dissolved in ethanol (1.5 mL) and Meldrum's acid (4.5 mg, 1.2 equiv.) and an aqueous solution of sodium carbonate 10% (160 µL, 6.0 equiv.) were added. The mixture was stirred at room temperature for 48h. A saturated solution of sodium hydrogenocarbonate (5 mL) was added and the organic phase was extracted with

dichloromethane (2 x 3 mL). The combined organic phases were washed with water (2 mL) and a saturated solution of sodium chloride (2 mL). The organic phase was dried with sodium sulfate and the solvent was evaporated. Compound **s3** (9 mg, 34 %) was obtained as a yellow paste. **s4** was observed at the major compound by LC-MS analysis (Figure S7) in a mixture with the starting material **s1**. Both compound could not be separated.

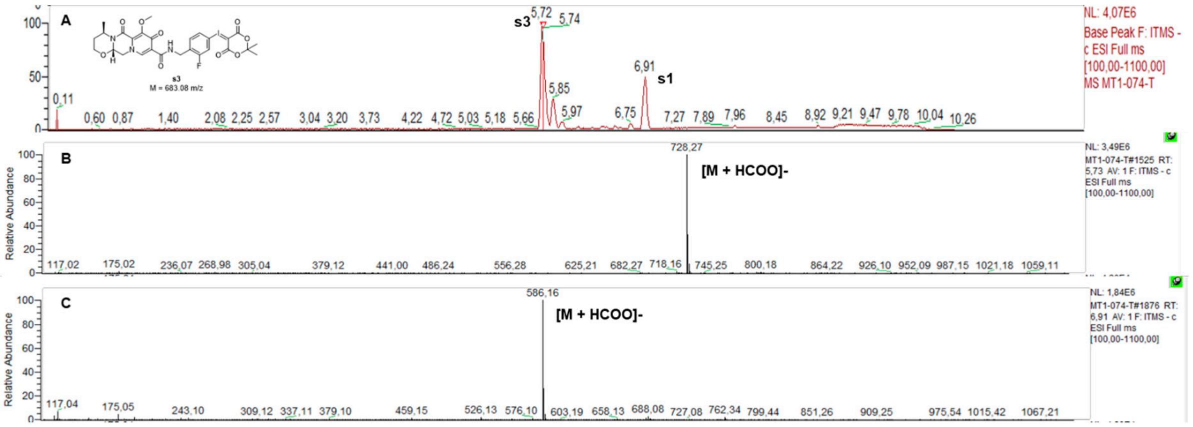

Figure S7. LC-MS$^-$ analysis of crude compound **s3**. A) LC analysis with MS$^-$ detection; B) Negative mass spectroscopy of the s3 peak at t_R = 5.72 min; C) Negative mass spectroscopy of the **s1** peak at t_R = 6.91 min.

(4R,12aS)-*N*-(2-fluoro-4-(4,4,5,5-tetramethyl-1,3,2-dioxaborolan-2-yl)benzyl)-7-methoxy-4-methyl-6,8-dioxo-3,4,6,8,12,12a-hexahydro-2H-pyrido[1',2':4,5]pyrazino[2,1-b][1,3]oxazine-9-carboxamide (s4)

To flame-dried potassium acetate (12 mg, 3.0 equiv.) was added a solution of **s2** (20 mg, 1.0 equiv.) in 1,4-dioxane (2 mL) under argon. $B_2(pin)_2$ (11 mg, 1.1 equiv.) and $PdCl_2(dppf)_2$ (2 mg, 0.1 equiv.) were added and the solution was stirred at 80 °C for 20h. Upon cooling to room temperature, water (2 mL) was added and the organic phase was extracted with dichloromethane (2 x 2 mL). The combined organic phases were washed with a saturated solution of sodium hydrogenocarbonate (2 mL), water (2 mL) and a saturated solution of sodium chloride (2 mL). The organic phase was dried with sodium sulfate and the solvent was evaporated. Crude compound **s4** (16 mg, 76 %) was obtained as a yellow paste. **s4** was observed at the major compound by LC-MS analysis (Figure S8) but was too unstable to be purified.

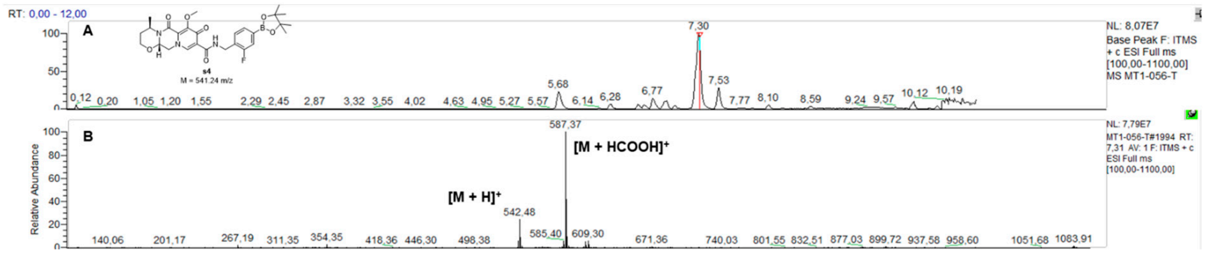

Figure S8. LC-MS$^+$ analysis of crude compound **s4**. A) LC analysis with MS$^+$ detection; B) Positive mass spectroscopy of the major peak at t_R = 7.30 min.

(4R,12aS)-*N*-(2-fluoro-4-(tributylstannyl)benzyl)-7-methoxy-4-methyl-6,8-dioxo-3,4,6,8,12,12a-hexahydro-2H-pyrido[1',2':4,5]pyrazino[2,1-b][1,3]oxazine-9-carboxamide (s5)

To a solution of compound **s2** (100 mg, 1.0 equiv.) in 1,4-dioxane (10 mL) and trimethylamine (2.0 mL) under argon were added $Pd(PPh_3)_4$ (45 mg, 0.2 equiv.) and $Sn_2(^nBu)_6$ (800 µL, 9.0 equiv.). The mixture was stirred at 110°C for 17h and the solvent was evaporated. The crude product was purified by flash chromatography on silica gel (dichloromethane/methanol) to afford **s5** (60 mg, 40 %) as a green paste. ^{1}H-NMR ($CDCl_3$, 400 MHz) δ 10.31 (t, J = 6 Hz, 1H), 8.35 (s, 1H), 7.30 (t, J = 7 Hz, 1H), 7.14-7.09 (b, 2H), 5.19-5.17 (b, 1H), 5.02-4.96 (b, 1H), 4.64 (d, J = 6 Hz, 2H), 4.22 (dd, J^2 = 13 Hz, J^3 = 4 Hz, 1H), 4.08 (dd, J^2 = 13 Hz, J^3 = 6 Hz, 1H), 4.00 (s, 3H), 3.94 (dd, J^2 = 9 Hz, J^3 = 2 Hz, 1H), 2.23-2.11 (b, 1H), 1.78 (s, 3H), 1.65-1.58 (b, 2H), 1.53-1.45 (b, 6H), 1.36-1.21 (b, 6H), 1.09-0.97 (b, 6H), 0.91-0.81 (b, 9H) ppm. ^{13}C-NMR ($CDCl_3$, 100 MHz) δ 174.5, 163.8, 155.7, 154.7, 142.1, 133.0, 129.1, 124.7 (d, J = 15 Hz), 122.5 (d, J = 17 Hz), 119.2, 76.2, 62.5, 61.1, 53.5, 44.5, 37.1, 29.4, 29.0 (3C), 27.9, 27.4 (3C), 26.9, 17.7, 16.1, 13.7 (3C), 9.7 (3C) ppm. HR-ESI(+)-MS m/z calcd for $C_{33}H_{49}FN_3O_5Sn$: 706.2673 $[M+H]^+$, found 706.2672.

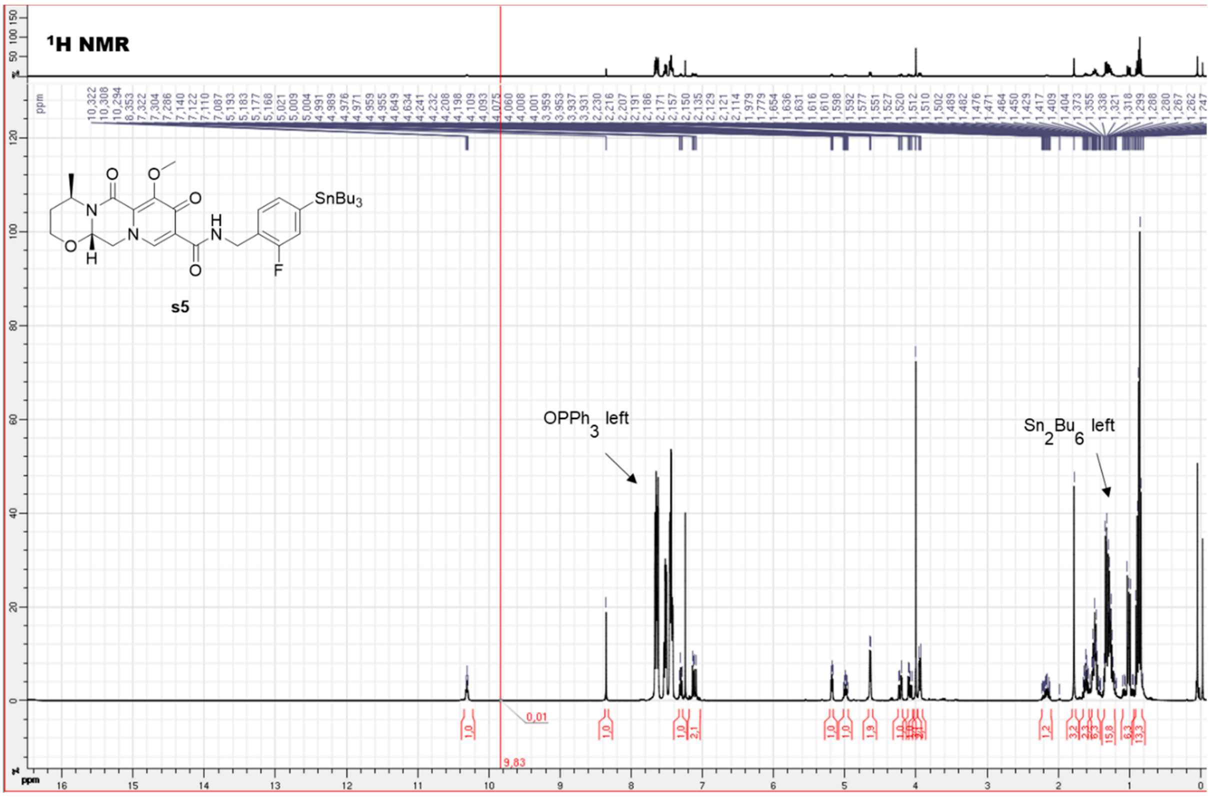

Figure S9. ^{1}H NMR of compound **s5**. Some $OPPh_3$ and Sn_2Bu_6 starting materials could not be separated from the compound.

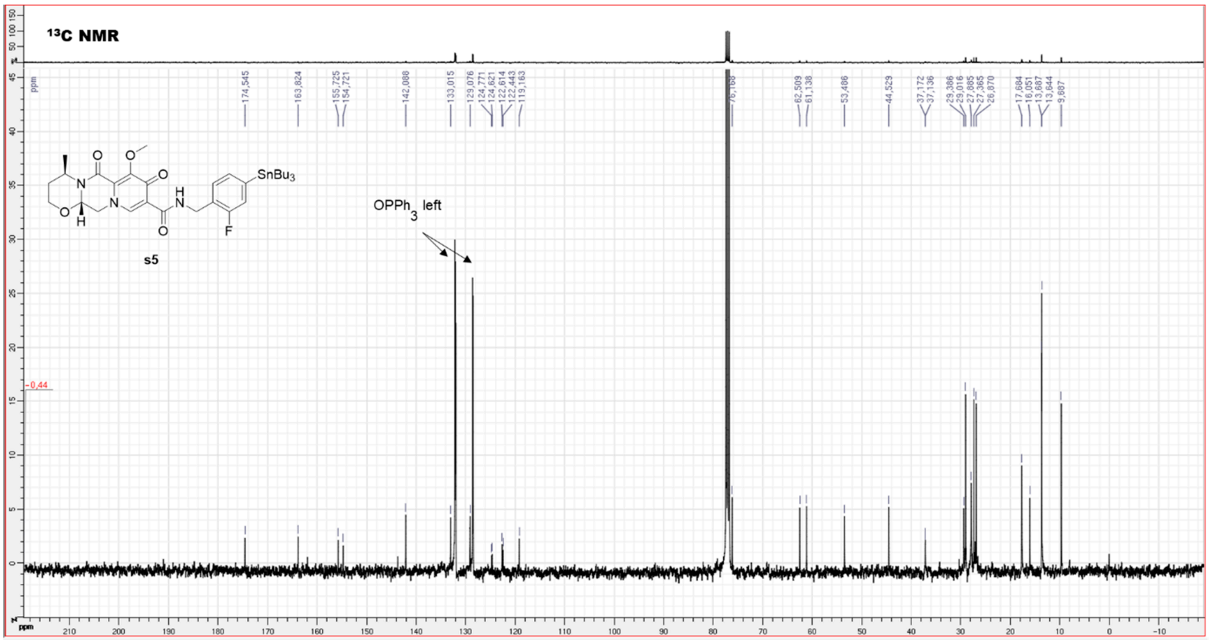

Figure S10. ^{13}C NMR of compound **s5**. Some $OPPh_3$ and Sn_2Bu_6 starting materials could not be separated from the compound.

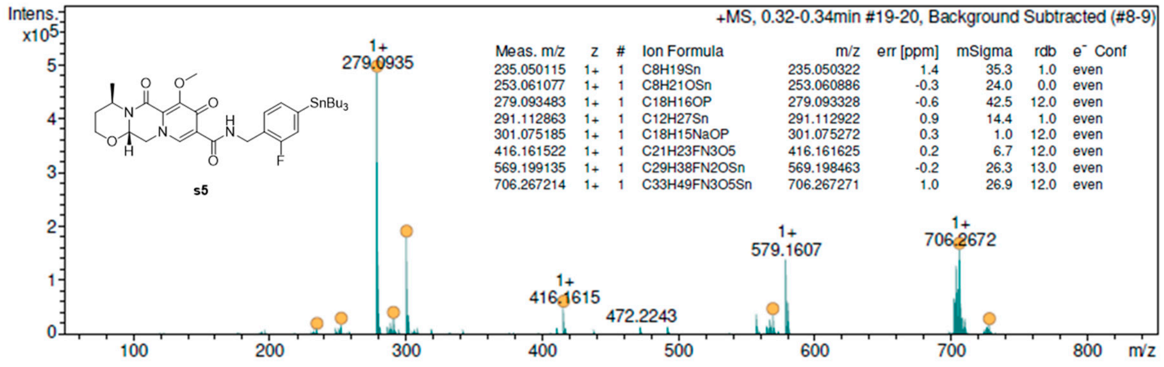

Figure S11. HRMS analysis of compound **s5**.

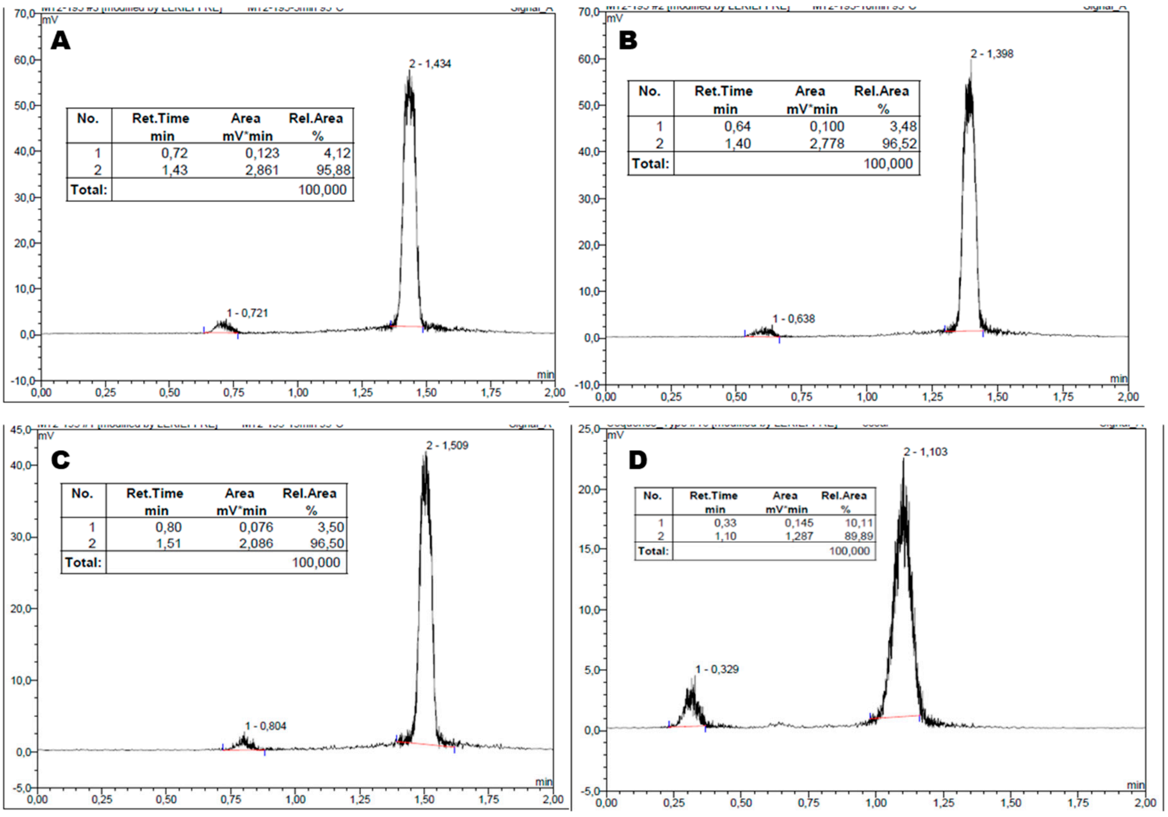

Figure S12. TLC analysis of the radiofluorination step. The first peak at 0.3-0.8 min is the unreacted [^{18}F]F$^-$ and the second peak at 1.1-1.5 min is the desired compound [^{18}F]**1**. A) Reaction in acetonitrile at 95 °C for 5 min; B) Reaction in acetonitrile at 95 °C for 10 min; C) Reaction in acetonitrile at 95 °C for 15 min; D) Reaction in DMSO at 95 °C for 5 min.

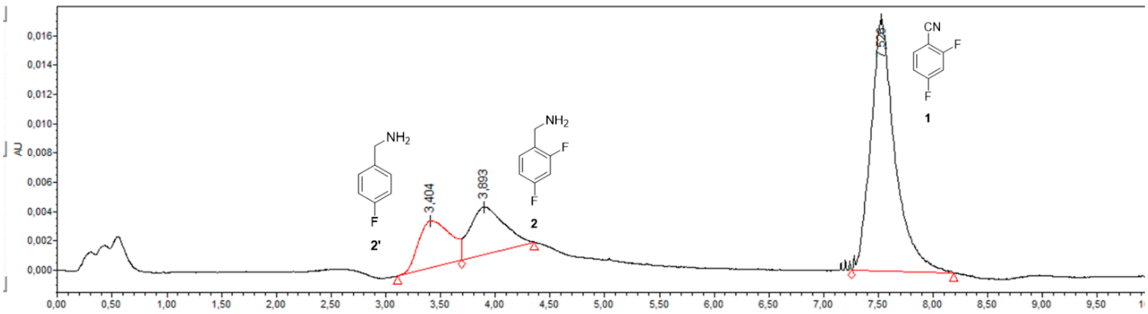

Figure S13. HPLC analysis of a mixture of compounds **1**, **2** and **2'**. Conditions: reverse phase analytical Symmetry® C18 (150 x 3.9 mm, 5 µm, Waters) column using a mixture of $H_2O/CH_3CN/Et_3N$ (80/20/0.1 v/v/v, 2 mL/min) as eluent and UV detection performed at 254 nm.

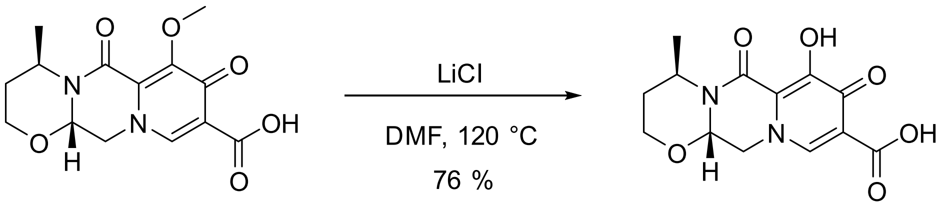

GSK3210346A **3**

Scheme S3. Synthesis of compound **3** from GSK3210346A.

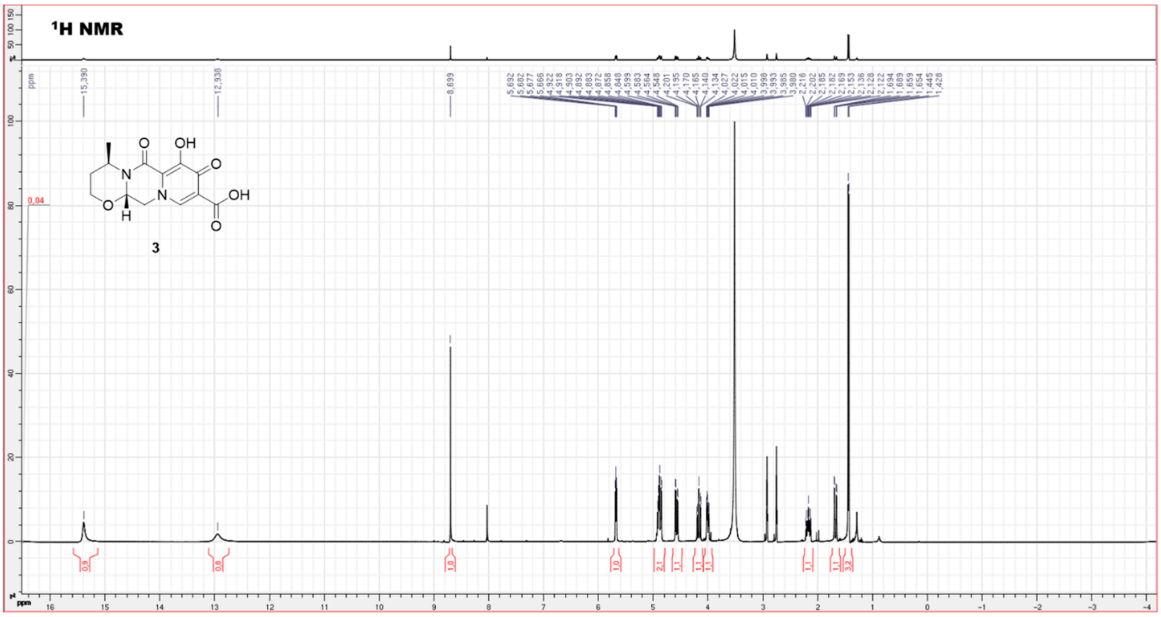

Figure S14. ^{1}H NMR of compound **3**.

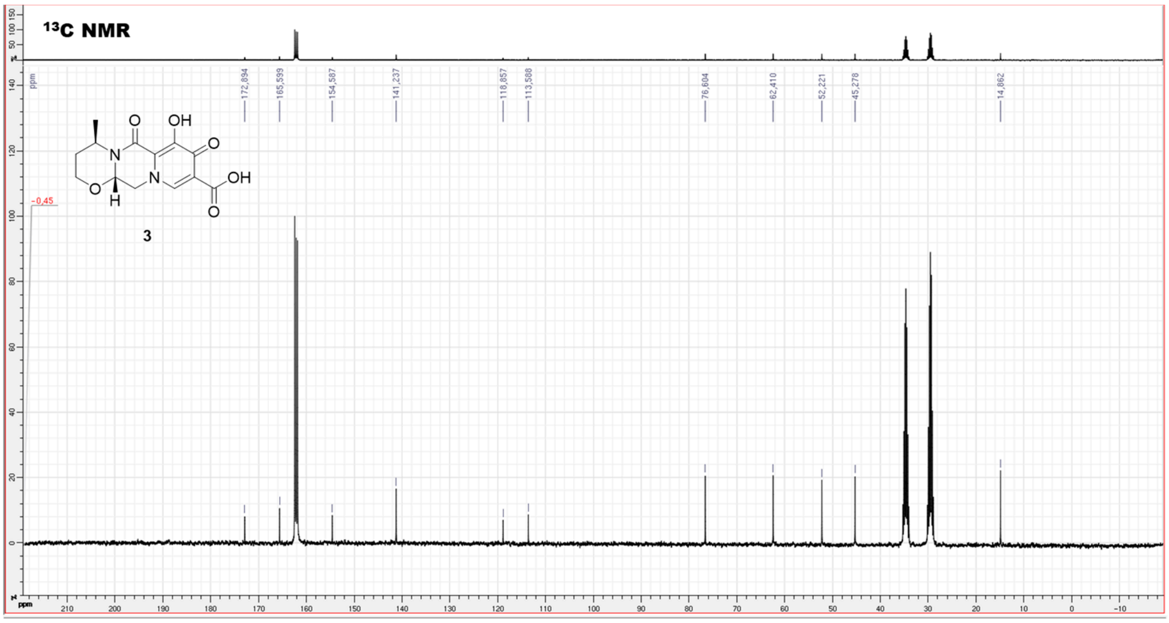

Figure S15. ^{13}C NMR of compound **3**.

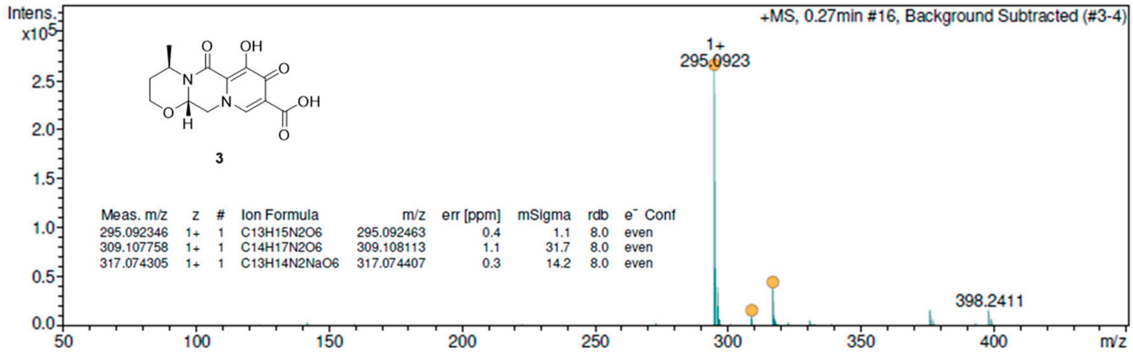

Figure S16. HRMS analysis of compound **3**.

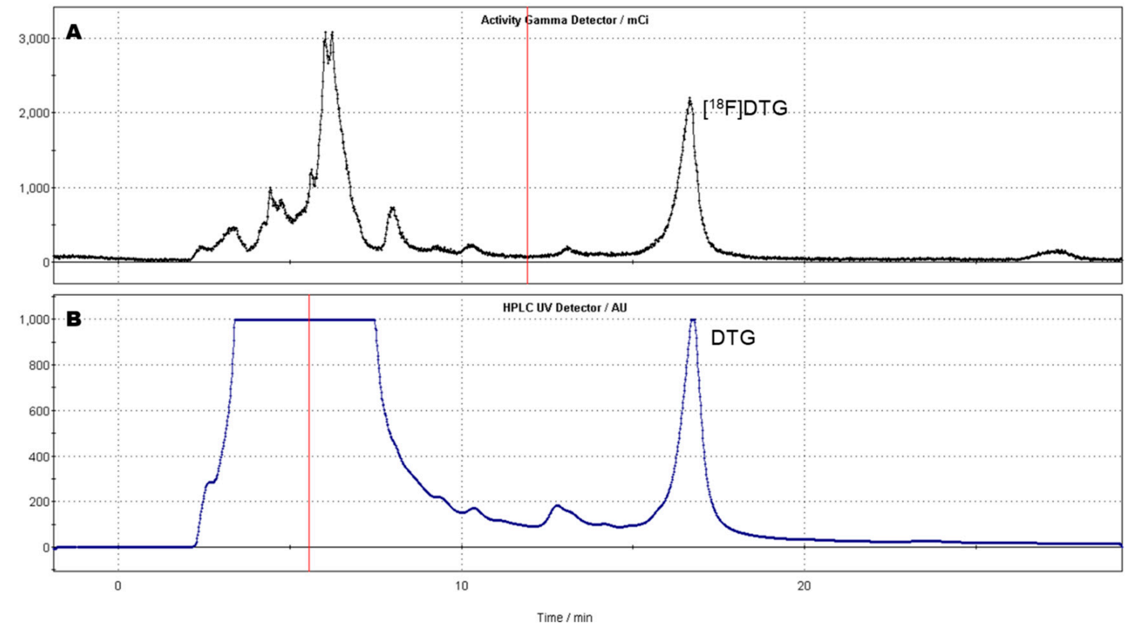

Figure S17. Semi-preparative HPLC purification of [^{18}F]DTG, which is obtained at a retention time of approx. 17 min. A) Gamma detection; B) UV detection at 254 nm.

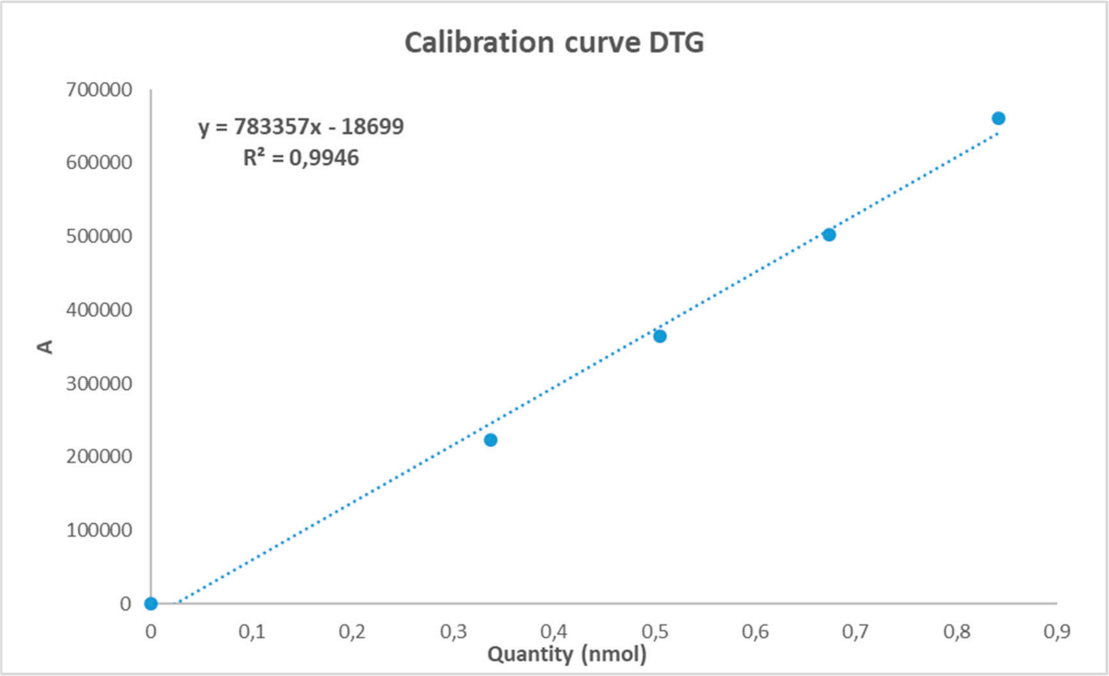

Figure S18. Calibration curve of DTG for molar activity calculation.
